# Trial experiences with dyadic breathalyzer technology for alcohol monitoring and feedback among couples living with HIV in South Africa

**DOI:** 10.1186/s13722-026-00659-1

**Published:** 2026-03-12

**Authors:** Amy A. Conroy, Rita M. Butterfield, Buyisile Chibi, Lindani Msimango, Tyrel J. Starks

**Affiliations:** 1 Department of Medicine, University of California San Francisco, San Francisco, CA USA; 2 Human Sciences Research Council, Centre for Community Based Research, Sweetwaters, KwaZulu-Natal, South Africa; 3 Department of Psychology, Hunter College of the City University of New York (CUNY), New York, NY USA; 4 Center for AIDS Prevention Studies, University of California San Francisco, 550 16th Street, 3rd Floor, San Francisco, CA USA

**Keywords:** Alcohol use, HIV/AIDS, Antiretroviral therapy, Couples, South Africa

## Abstract

**Background:**

Dyadic monitoring tools could enhance partner awareness of drinking behaviors and prompt timely communication and supportive responses during alcohol use. We posited that real-time blood alcohol content (BAC) levels combined with motivational interviewing (MI) could facilitate couple communication, communal coping, and support, enabling couples to set joint goals and reduce alcohol consumption. We qualitatively explored the positive and negative experiences of trial participants who received mobile breathalyzer technology with couples MI to reduce unhealthy alcohol use in South Africa.

**Methods:**

Thirty couples (60 individuals) participated in a pilot trial combining couples-based MI with mobile breathalyzers. Index participants reporting unhealthy alcohol use and on antiretroviral therapy were assigned a portable breathalyzer linked to a smartphone app to complete twice-daily BAC tests. Partners received real-time SMS notifications of these BAC results. Both partners engaged in three MI sessions to reflect on drinking patterns and set alcohol reduction goals together. Transcripts from audio-recorded sessions and exit interviews from a subset of 12 individuals were analyzed using framework analysis. The data were coded for positive and negative experiences of using the breathalyzers and dyadic mechanisms of behavior change.

**Results:**

Participants averaged 35.9 years old and had relationships averaging 6.6 years. Most couples (66.7%) had two partners reporting unhealthy drinking. Couples found the mobile breathalyzer technology engaging and enjoyable due to its novelty and social curiosity it sparked. Despite challenges related to connectivity issues, privacy, and technical difficulties, participants generally found workarounds and leveraged study resources like power banks and dedicated navigators. Participants described how the breathalyzers enhanced self-awareness of alcohol consumption, promoted home-based drinking, and fostered partner support and communication, though it sometimes led to tensions over technology allocation and usage, when only one partner received the device.

**Conclusions:**

Couples in a low-resource setting in South Africa successfully used breathalyzers to self-monitor alcohol consumption. Breathalyzers could be used to increase personal motivation to reduce alcohol use, activate partner support, and help partners coordinate efforts to achieve drinking goals. Addressing structural challenges like cellular connectivity, as well as dyadic equity by providing both partners with breathalyzers, will be critical to maximizing breathalyzer uptake and intervention impact in resource-poor settings.

**Trial registration:**

NCT05756790; registration date: 3/6/2023.

## Background

In sub-Saharan Africa (SSA), alcohol use is a major impediment to HIV virologic control. Unhealthy alcohol use (AUDIT-C score greater than 3 or 4) is common among people living with HIV (PLWH) [1]. Worldwide, South Africa has among the highest levels of per capita alcohol use (liters of pure alcohol per year per person) and heavy episodic alcohol use (6 + standard drinks on a single occasion) [2]. Among PLWH who drink in South Africa, an estimated 50–90% engage in unhealthy alcohol use [3–5]. Alcohol use contributes to suboptimal adherence to antiretroviral therapy (ART) and poor retention in HIV care in South Africa [6–8].

For PLWH in relationships, alcohol use may be best addressed through couple-level interventions. Unhealthy alcohol use is correlated with poor couple communication [9] and conflict [10], and alcohol use can also undermine ART adherence by weakening couple relationships needed for support, daily functioning, and well-being [11, 12]. Dyadic interventions can target relationship dynamics linked to alcohol use and efficiently address both partners’ unhealthy alcohol use. Starks developed a novel approach for addressing substance use and HIV risk in couples referred to as couples motivational interviewing (MI) [13–15]. Evidence suggests indicates that couples MI is a feasible, acceptable, scalable, and effective strategy [16, 17]. Other couple-based interventions using MI techniques have shown promise in decreasing alcohol use and improving relationship dynamics in couples living with HIV in the U.S [18].

Couples MI interventions incorporate substance use feedback and debriefing activities into sessions, however, none have integrated daily monitoring and biological-based feedback. A meta-analysis found that MI with individuals was more effective at reducing alcohol consumption when personalized feedback was included in sessions [19]. Digital methods for monitoring and feedback show promise. The *Health Call* study found that combining MI with smartphone-based monitoring and feedback resulted in a greater reduction in drinking days than MI alone [20–22]. The Tracking and Reducing Alcohol Consumption (TRAC) study examined the feasibility and acceptability of using mobile breathalyzer technology for alcohol monitoring with PLWH in the U.S. who reported unhealthy alcohol use [23]. The testing protocol was well-tolerated with 80% of breathalyzer tests completed, and participants expressed favorable views toward the breathalyzers [23, 24].

For PLWH in relationships, dyadic monitoring tools could enhance partner awareness of drinking behaviors, and prompt timely communication and support. Per interdependence theory [25], we posit that shared information on a partner’s drinking levels – when combined with couples MI – could help to spur communication, communal coping around alcohol use, and social support so that couples would work together to set goals and reduce one or both partners’ drinking. Devices like the BACtrack^®^ View breathalyzer have been designed for use with partners. BACtrack^®^ View sends a designated person (tester) an SMS request to complete a BAC test via a mobile app with breathalyzer. Designated primary partners receive a message with the tester’s BAC result immediately following the test. The BAC data can be summarized onto a calendar within the MI sessions and used to encourage reflection on drinking levels and joint goal-setting.

No studies have utilized the breathalyzer technology for dyadic feedback, and thus little is known about couple experiences using breathalyzers to trigger social support around alcohol reduction. Moreover, most studies involving breathalyzer monitoring have been conducted in the U.S. or other developed countries [26–28], and their applicability in low-resource settings remains unclear. Factors such as technology literacy, internet connectivity, stigma around alcohol use, and social or cultural norms—particularly regarding the sharing of private test results within couples—may vary significantly across contexts. In this qualitative study, we examined exit interview data from couples who received mobile breathalyzers with dyadic feedback and couples MI as part of a randomized controlled trial in KwaZulu-Natal, South Africa (*Masimbambisane* study*)* [29, 30]. We examined positive and negative experiences using the breathalyzer technology and identified ways in which couples engaged with the technology and BAC results to change drinking behaviors.

## Methods

### Study overview

*Masibambisane* took place in Sweetwaters, a rural community to the west of Pietermaritzburg, in KwaZulu-Natal province, South Africa. South Africa is a middle-income country in sub-Saharan Africa with high smartphone penetration rate (over 68%) as compared to other African nations [31], making it an suitable context to pilot test a mobile breathalyzer technology. Ninety couples were randomized to one of three arms: enhanced usual care (EUC; control arm), couples MI (MI-only), and couples MI plus mobile breathalyzer technology (MI-plus). For the current study, we conducted exit interviews with a subset of trial participants in each arm.

To be eligible, couples were required to be: (1) in a primary relationship for at least six months; (2) aged 18–49 years (both partners); (3) have at least one partner (the “index patient”) with a positive AUDIT-C screen (score ≥ 3 for women and ≥ 4 for men) and on ART for at least six months. Primary partner was defined as “a person who you are committed to above anybody else and with whom you have had sexual relations”. Participants living with HIV were required to have disclosed their HIV status to their partner. We excluded those who feared for their safety and/or reported severe IPV in the past 3 months based on the World Health Organization (WHO) measure of domestic violence [32].

Couples were recruited using a community-based approach to identify participants at community-based organizations or at drinking establishments such as shebeens or bottle stores. Flyers were also placed and distributed in the community and clinics. Refer to the study protocol for details [30].

### Intervention arms

Couples in the MI-only and MI-plus arms received three, in-person couples MI sessions with a trained counselor in their preferred language (*isi*Zulu or English). Details on the intervention are described elsewhere [29, 30]. Three sessions took place a month apart over a period of 2 months. MI sessions were conducted with both partners together and each lasted 60 to 75 min.

For the MI-plus arm, index participants completed twice-daily BAC tests (11 am and 8 pm) using a mobile breathalyzer and app for 60 days. The partner received the twice-daily SMS messages with index participant’s BAC result in real-time. We scheduled tests (versus random tests) per the recommendations of our team and focus group participants [30]. Scheduled tests allowed participants to plan that their devices were charged and cellular connectivity was available. To offset the chance that participants could skip a test with an anticipated high reading, a small incentive was provided for tests completed. Smartphones were provided to index participants in the MI-plus arm. At the start of the study, the technology was only available for IOS, and thus refurbished iPhones were provided to a subset of couples (*N* = 19). Halfway through, the technology became available for Android – a common smartphone in this setting. Prior to launch, couples were trained on how to operate the mobile breathalyzer and app, followed by a short trial period. Index partners were provided with monthly data bundles to ensure continuous access to the breathalyzer app. For couples where two partners reported unhealthy alcohol use, the male partner was selected for the breathalyzer given that unhealthy alcohol use is more severe among men [33]. BAC test data was overlaid onto the monthly calendars in MI sessions, presented to the couple for reflection, and used to trigger discussions about joint goal-setting.

Feasibility and acceptability results are presented in the trial paper [29]. For the MI-plus arm (29 couples; 58 people), 58.2% of all BAC tests were completed [29] and 72.4% of participants completed at least one BAC test per day. When participants completed a test, the average time to take the test (within-person) was within 20 min of receiving the request. Around 93% reported being satisfied with the breathalyzer technology [29]. Trial results also showed greater (but not significant) declines in AUDIT-C score among those in either treatment arm as compared to control. However, there were significant effects on AUDIT-C scores at follow-up for both treatment arms among those reporting severe/high risk drinking at baseline [29].

### Data collection and procedures

To contextualize the trial data, we conducted exit interviews with 15 couples (30 people) after the 2-month visit. Participants were recruited from each arm, with an oversampling of couples from intervention arms. We purposively sampled a mix of couples with varying session attendance rates (i.e., attended one session versus all) and breathalyzer completion rates (i.e., completed some tests, or most tests). To obtain a range of experiences, we also included couples having one and both partners with unhealthy alcohol use, as well as both sero-concordant and discordant couples.

Trained, gender-matched interviewers with prior qualitative skills conducted the exit interviews in participants’ preferred language. Each partner was interviewed separately, but simultaneously, in private rooms to ensure confidentiality and independent responses. Interviews lasted 60–90 min. We elicited information on satisfaction with the intervention (e.g., frequency of breathalyzer tests and MI sessions, length, content). To explore dyadic mechanisms of change, we elicited information on how breathalyzers were used within the couple and how the breathalyzers plus MI enhanced communication, communal coping, and support. We also elicited information on technology issues (e.g., user experience, network connectivity, power outages, battery life, trouble with logins) and contextual issues (e.g., literacy, alcohol stigma). Given the central question of whether the mobile breathalyzer technology could trigger support, we asked questions around how knowledge of BAC levels impacts the relationship (e.g., increases in conflict, communication), how both partners interpreted and responded to BAC results, and the types of partner support provided. Interviews were audio-recorded, transcribed word-for-word, translated into English, and spot-checked for quality.

As part of fidelity procedures for the trial, we audio-recorded MI sessions which included discussions of the BAC results using a calendar activity. Given the large number of recorded sessions, we transcribed and translated MI sessions from a subsample of 14 couples (up to three sessions per couple). Couples were purposively selected to balance high participation, low participation, dual-drinker couples, and single-drinker couples.

### Analysis plan

The final sample consisted of MI-plus couples who completed an exit interview (12 participants in 6 couples). Of these couples, four couples also had session transcript data that was included. Three out of 4 of couples had 3 sessions available for analysis; one couple only had one session available (only attended one MI session). Thus, the final dataset consisted of 22 transcripts from either interviews or sessions (see Table 1).

**Table 1:** Baseline Characteristics of the Qualitative Sample from the Masibambisane Study

ID	Includes MI session data	Gender	Study arm	HIV status	Session completion rate	BAC test completion rate	Dual drinking couple	Relationship length	Age	AUDIT-C score	Married	Cohabitation status
8_I	X	Male	MI+	Positive	33	58	Yes	4.46	37	8	No	Yes
8_P	X	Female	MI+	Positive	33	NA	Yes	4.46	35	9	No	Yes
9_I	X	Male	MI+	Positive	100	83	No	1.37	37	7	No	No
9_P	X	Female	MI+	Positive	100	NA	No	1.37	26	2	No	No
13_I		Female	MI+	Positive	100	23	Yes	0.36	33	5	No	No
13_P		Male	MI+	Negative	100	NA	Yes	0.36	32	8	No	No
69_I		Male	MI+	Positive	100	53	Yes	5.08	41	8	No	Yes
69_P		Female	MI+	Positive	100	NA	Yes	5.08	44	7	No	Yes
50_I	X	Male	MI+	Positive	100	75	No	20.97	43	8	No	Yes
50_P	X	Female	MI+	Negative	100	NA	No	20.97	49	2	No	Yes
101_I	X	Male	MI+	Positive	100	89	Yes	11.65	35	7	No	Yes
101_P	X	Female	MI+	Positive	100	NA	Yes	11.65	30	7	No	Yes

NA = Not Applicable; EUC=Enhanced Usual Care; MI=Motivational Interviewing; BAC=Blood Alcohol Content

We used framework analysis to examine the experiences of couples. Starting with the exit interview transcripts, content related to use of the breathalyzers or phones was labeled with broad code (“breathalyzers and phone”), which was added to a Microsoft Excel worksheet. Columns were created within the worksheet for codes with corresponding raw quotes. Codes (i.e., columns) within this sheet included “dislikes,” “likes”, “challenges with breathalyzers,” and “how breathalyzers changed alcohol use”. Within each column, we systematically categorized emerging themes, developing inductive codes based on participant narratives. For instance, within the “challenges with the breathalyzer” category, discussions surrounding connectivity issues led to the creation of the “network and power” sub-code. The data was organized such that each row consisted of a participant. Consistent with our published dyadic analysis approaches [34–36], partners of a couple were grouped together.

Session transcripts were coded using the same codes for the interview data. Additional rows were added to the Excel worksheet for each session transcript. A team of five coders were given a set of exit interview transcripts to code (3 students, the lead author, and 3 project managers). For the session transcripts, there were 4 coders (2 students, 1 project manager, and the lead author) including representatives from South Africa and other African settings. The lead author trained the coders, developed the codebook, performed quality assurance on coding, and lead the thematic analysis. Team meetings were held to review the codes and coding process, and to discuss emerging themes.

Next, the lead author grouped the data into inductive themes with associated quotes using Microsoft Word. To assist with the presentation of themes and findings below, we used Versa Chat to draft the initial narrative. Versa Chat is a HIPAA-compliant web interface developed at the University of California San Francisco (UCSF) that allows users to engage in interactive conversations with large language models such as Azure OpenAI’s GPT-4o. As an extra layer of protection, de-identified data were further anonymized to remove names, places, or other identifiers. Prompts were given to describe the study with our published protocol, to instruct Versa Chat to refer to published manuscripts for tone and writing style, and to provide formatting instructions (e.g., italicize quotes, etc.). The output was compared against the Microsoft Word themes and additional prompts were used to generate narratives for sections of codes that were missed. The narrative and themes generated by Versa Chat mirrored our identified theme names—for example, “dyadic imbalance” emerged from codes and quotes related to relationship dynamics and perceptions of fairness.

The lead author conducted a rigorous verification process to ensure that AI-generated text accurately reflected the underlying subthemes and participant narratives identified in manual coding. Through this iterative approach, we refined the thematic structure and edited the narrative substantially, ensuring fidelity to the original data and lived experiences. Additional edits were made by the research team in South Africa who provided cultural expertise.

## Results

### Sample characteristics

Demographic data were collected at the baseline visit from index participants who were assigned to the breathalyzer and their partners. Refer to Table 1. The average age was 35.9 (range: 30–49), and couples were together for an average of 6.6 years (range: 8 months to 21 years). Four of six couples (66.7%) were classified as dual heavy drinkers. Regarding intervention engagement, only one couple did not complete all three MI sessions (16.7%). An average of 63.5% of BAC tests were completed. Five out of six index participants who were assigned to the breathalyzer technology were male.

### Positive experiences with the mobile breathalyzers

#### Technology as a source of enjoyment and learning

Many described how using mobile breathalyzer technology was engaging and enjoyable, bringing a sense of novelty and social curiosity from others. Some participants were intrigued by the technological features, such as the breathalyzer’s speed and the app’s ability to track their location which made them feel connected. A male index participant who received the technology plus the MI sessions remarked how he ranked the technology and the phone as his favorite aspect of the study:

*What I liked the most is the phone…And taking the tests—it didn’t bother me because I enjoyed it. There was so much joy; I was happy.* (male, index partner, age 41, couple 69)

While learning to use the breathalyzer and phone required patience and confidence in one’s capabilities, participants felt that the level of learning required was acceptable and not a significant barrier. Another male participant reported:

*The breathalyzer and the phone need you to be patient and give them time…it’s not a difficult thing, you just have to tell yourself that you can.* (Male, index partner, age 37, couple 9)

#### Embracing the breathalyzer in public spaces

Participants reported feeling comfortable using the breathalyzer device and app in public spaces, such as drinking establishments, and easily incorporating the intervention into their daily lives. Some people found the handheld breathalyzer with app easy to carry and use – at least at drinking places and around other people who drink. As one man described:

*I was happy to carry the phone with data…I would carry my phone and breathalyzer to a shebeen, so when it was time, I would find the right spot with light and take my test.* (male, index partner, age 37, couple 8)

While using the breathalyzer in public sometimes invited curiosity from others, participants were generally unbothered by the questions and did not find this to be a significant barrier to using the breathalyzers. One man described how he had no problems with the device, but described feeling compelled to answer others’ questions about it.

#### Study resources enhanced technology engagement

Participants described how the technology navigators, who provided regular phone calls or in-person visits, helped to ease technological barriers. Participants also appreciated having a power bank that could be used to charge the phones and breathalyzers, ensuring that a low battery was never an excuse for missing a test. Additionally, providing cellular data made it easier to complete tests since participants did not have to pay for the data. Participants noted that the data supply was consistent, and if it ran out, it was replenished by the study team. Finally, financial incentives for completing each breathalyzer tests also played a motivating role. Some noted how even a small reward was enough to reinforce engagement:

*I was getting paid, which I liked because when you inhaled/pumped, there was R5 you received.* (male, index, age 43, couple 50)

While not all participants received an iPhone, those that did, developed a deep attachment to the device – valuing both its status and functionality. The phone’s appeal extended beyond index participants, with partners also reporting benefits from the cellular data provided by the study. For some, using the iPhone became so ingrained in their daily routine that transitioning back to their personal phones was difficult after the study ended.

### Challenges and frustrations with the mobile breathalyzers

#### Privacy and social stigma: “What is this thing you are doing?”

Contrary to the participants above who embraced the technology in public spaces, others reported how using the breathalyzer in public spaces or social events felt awkward and intrusive. They were met with skepticism and sometimes even resistance from people around them. One male index described how some people thought he was sick (thinking the device was an inhaler) and others thought he was capturing their photo without permission when completing a BAC test:

*I would breathalyze in front of other people… And they were looking at me like I am crazy, saying, ‘Hey, what is this thing you are doing?’ One person even said, ‘Get away from me, you will give me a chest infection…’ Another would say, ‘Why are you taking a picture of me?* (male, index, age 35, couple 101)

Because of this discomfort, some chose to avoid public breathalyzing altogether, restructuring their testing to ensure privacy. Another man became resigned to using the device in the privacy of his home, due to negative reactions from others:

*That’s the reason that encouraged me to stay at home… Yes. The rules were that you don’t do it at the tavern and not in front of people. I used to make sure I’m away from people most of the time.* (male, index, age 33, couple 45)

Carrying the device to social gatherings also posed an issue for those who were accustomed to a more carefree lifestyle and found it inconvenient, as one man reported:

*What I can raise is that since we are the kind of people who party – I would sometimes find it difficult to carry it.* (male, index, age 35, couple 101)

#### Network and power struggles: The largest barrier to testing

One of the most common barriers reported by participants was having unreliable cellular network connectivity, which interfered with the ability to complete breathalyzer tests. One couple told their MI counselor:

*We experienced network problems that even affected our ability to log in with the email address. The network was quite troublesome.* (couple 8, MI session 2)

Some participants noted that network problems weren’t consistent—sometimes, connectivity would work fine in certain locations but fail in others. One male index described:

*It was mainly an issue in the area. There were times when they came to check [the technology navigators] and found that I had sufficient data, but it still wasn’t connecting. However, when they connected their phone, it worked fine. I even found better connectivity in another tavern nearby, or take the test outside on the road.* (male, index, age 37, couple 8)

Power outages added another layer of difficulty, further disrupting connectivity. In some cases, power banks and cellular data provisions were insufficient. If power went out, cellular service was often out too – as described by the female partner below. Our research team in South Africa confirmed that battery packs – necessary to keep cellular service functioning during periods of load shedding – were often stolen from the cellular towers to sell off their parts.

*Yes, it was trouble when he had to do the breathalyzer where he was; you find that when there is no electricity, even there is no network.* (female, partner, age 35, couple 8)

For some, these repeated connectivity failures led to missed tests, even when they made efforts to comply with the protocol. A female partner explained the dilemma:

*The outage would perhaps be from 6:00 PM, and it would be back on at 9:00 PM. Then he would have to breathalyze at 8:00 PM, and there would be no network connectivity. Yes…And he would end up not breathalyzing then.* (female, partner, age 26, couple 9)

However, others developed workaround strategies by visiting locations they knew had connectivity, such as outside the home or a location nearby. In some cases, network failures caused frequent delays requiring external assistance to resolve. This could require a home visit by the technology navigator to help solve the issue, as described by this male index:

*It’s just that sometimes it refused to connect, so that used to disturb me because then I wasn’t able to get a reading until I got [name of research assistant] the following day to assist me. Usually at night, and then I had to wait until the morning to fix it.* (male, index, age 37, couple 9)

#### Technical and usability issues

Beyond network and power challenges, participants faced additional hurdles related to the breathalyzer app itself. Malfunctions and erroneous readings could make breathalyzing an unpredictable experience. However, in the case below, challenges experienced could have been due to connectivity rather than the device. It is not possible to tell from the interview.

*The location was turned on; everything was turned on—Bluetooth and everything. But I would do the test, and it would keep on loading, loading, loading, loading. And then it would cancel and then cancel again.* (male, index, age 35, couple 101)

There were also instances where participants were unclear how to interpret their BAC readings, leading to confusion about the results. This was potentially due to health literacy issues than the device, suggesting a need for more explanation around BAC levels. A male index described the confusion:

*The issue is that we have no idea how to determine whether anything is at the top or bottom of the scale. That’s the problem we face. We’re uncertain whether a reading like 0.000 indicates a high or low level, as it’s often the result we see.* (male, index, age 35, couple 101)

In other cases, an erroneous reading conflicted with explanations on BAC levels provided during the intervention. One particularly concerning challenge was when participants saw unexpectedly high BAC readings despite consuming little alcohol, making them doubt the accuracy of the device. As one male index explained:

*Yes, sometimes, especially when I saw high percentages indicating I was heavily intoxicated on days when I hadn’t consumed much alcohol.* (male, index, age 37, couple 8)

The technology onboarding session provided training on scenarios that could lead to unexpected readings (e.g., use of hand sanitizer); however, confusion remained among participants. Other practical issues—such as using up cellular data required to complete tests, faulty or lost chargers, and forgotten passwords—were additional sources of frustration.

#### Relationship strains: Dyadic imbalances and conflict

The intervention design involving the allocation of breathalyzers also led to unexpected tensions within some couples. Some partners (mostly women) felt excluded because only one person in the relationship received the phone and breathalyzer. For some, the disappointment of not receiving the technology created initial resentment:

*At first, I was heartbroken because I thought only he would be receiving a phone. And I looked at the phone, and it was such a beautiful phone… But ultimately, I ended up supporting him.* (female, partner, age 26, couple 9)

It was rare for the intervention to induce couple conflict. However, the introduction of the iPhone fueled an argument in one couple. In this case, the female partner believed her partner was using the phone to court other women:

*It used to annoy me. It’s because he started acting sly! Because he was carrying an iPhone. I now find pictures of females, and when I ask, he would say, ‘Isn’t it that I carry a nice phone, so the person asked me to take pictures of them because this phone takes nice pictures.’ But why are you keeping them?* (female, partner, age 35, couple 8)

### Mechanisms of behavior change induced by the breathalyzers

#### Transition to home-based drinking

Some participants reported a significant shift in their drinking habits, by opting to spend more drinking time at home than at taverns. Participants found it challenging to maintain the required connectivity in taverns and bars, which often led to missed tests. This connectivity issue made it more practical to stay at home, where they could reliably perform tests. As described by one male index:

*The problem was that if the connection was lost, I used to forget to do it again, especially when I went to the tavern…That’s what made me reduce going to the tavern too often because I had to wait for the time together with my partner.* (male, index, age 26, couple 9)

Participants expressed that being home for breathalyzer tests created a sense of responsibility and curbed late-night outings, which were associated with heavier drinking. In an MI session, a couple explained to the counselor how the male partner came home early to test:

*Counsellor: What was helpful when you were using the breathalyzer? Male Partner: What was helpful was the fact that if I’m out, I know that maybe by eight (8) o’clock I must be at home. It limits me from going until late.* (couple 50, MI session 3)

Moreover, the potential risk of losing or damaging the device while intoxicated prompted them to manage their alcohol intake more carefully. One male index participant described:

*Yes, and I knew I should not get too drunk because I was carrying a phone and a breathalyzer. Yes, I needed to be careful and not drink much and go home. The phone helped me a lot because I wasn’t drinking as much as I used to.* (male, index, age 35, ID couple 8)

#### Enhanced self-awareness and setting limits

The immediate feedback provided by the breathalyzer allowed individuals to see the direct impact of their drinking habits, which led to self-regulation and reduction in alcohol consumption. While the breathalyzer did not provide any “warnings”, participants interpreted the BAC readings as cautionary signals to reduce drinking levels. One male index described:

*The breathalyzer used to warn me that I should limit it as I had drunk too much…And also breathalyzing was helpful because one could keep track of how much alcohol one had consumed.* (male, index, age 43, couple 50)

For one man, receiving a lower BAC result helped to reinforce positive change:

*What I like is taking the test and seeing the level of drinking, how it had dropped, and the days. I like that a lot. Because it made me see that I had sinned and then I reduced.* (male, index, age 41, couple 69)

#### Partner dynamics and support around breathalyzer use

The involvement of partners in the breathalyzer process played a significant role in influencing drinking behavior, with multiple forms of partner support described. Partners often took on roles such as reminding the participant to take the test, managing the breathalyzer device, and even assisting with the technical aspects like logging into the app. One man described how his partner provided reminders to test, monitored his drinking, and disciplined him if needed:

*It was okay because she would watch over me. If it was time for the test and I was away from her, she would call or send an SMS to remind me. We both planned on how we would share the test money every month. That is why she was watching me. We got close like that because we guided each other. If I was misbehaving with the phone, I would give it to her.* (male, index, age 37, couple 8)

This cooperation extended to managing the technology, sharing financial responsibilities related to the breathalyzer, and providing support. The following male index did not mind the monitoring and reminders from his partner, which he described as necessary to keep him in line.

*Participant: She would tell me that I overdrank. Interviewer: So, it spied on you. Participant: Yes, it did, but it wasn’t a big deal because it was disciplining me. Yes, she supported me a lot. Even when it came to taking a breathalyzer, I used to do it often because of her… She used to remind me; she was my reminder when it was time to take the test. She would stop me from leaving and tell me that by 9, I should be home.* (male, index, age 37, couple 9)

In addition to reminders to test, some partners helped with logins and managing passwords, ensuring that the index participant could complete the tests without technical difficulties.

The breathalyzer results could serve as a basis for communication between partners about their drinking habits. This open dialogue allowed couples to discuss their alcohol consumption levels and provided a platform for mutual guidance and encouragement to reduce drinking. A female partner talks about how they set goals to reduce weekend drinking after receiving a high BAC result:

*Oh no, now it has become too much [referring to BAC value]; let us reduce… We would reduce – did you see that in this month it seems there were many days wherein we consumed alcohol? No way. We did not skip even one weekend…That is what you would find us discussing – that there ought to be a weekend that we skip and not drink. It helped us a lot.* (couple 50, MI session 3)

Some couples used the breathalyzer data to engage in reflective conversations about their drinking patterns, which helped them set boundaries to reduce their alcohol intake.

*Counsellor: [Inaudible 08:15]. Okay, have both of you ever discussed the readings when you see the BAC levels? Female Partner: Yes, we usually discuss them and note the many spaces [days]that have been missed / skipped.* (couple 101, MI session 3)

However, not all couples talked about the BAC levels and some admitted that alcohol was not an issue for them, thus there was no reason to discuss:

*Counsellor: Did you talk about the BAC level? Female Partner: No, we don’t talk about that. Because I think we have that understanding where the alcohol doesn’t bother us so as a couple…So, even if its high or even if its low, we don’t say anything, we just [crosstalk – 41:03]. Male Partner: We just check and its done.* (couple 50, MI session 2)

In a few couples, financial coordination also emerged as a theme, where couples discussed and made joint decisions about their spending, with some noting a reduction in money spent on alcohol as a positive outcome of using the breathalyzer.

*It helped us because we were even wasting money. We used to come back from town and find that every weekend I am drinking, we are drinking because we were both drinking. I could say it helped me a lot because we have now reduced. We know that when we have money, let’s start by doing important things and end with the less important things.* (female, index, age 41, couple 69)

In other cases, partners would encourage BAC testing and provide reminders to index participants so they could maximize financial benefits from the testing incentive. However, some participants expressed disappointment around how only one partner received the testing incentives despite their role in providing social support around breathalyzer testing.

## Discussion

These findings broadly suggest that couples, even in a low-resource setting in South Africa, were able to successfully use breathalyzers to self-monitor for alcohol consumption. For many couples, doing so increased personal motivation to reduce alcohol use, activated partner support, and helped both index and recruited partners to coordinate efforts to carry out drinking goals. Although some challenges were noted, particularly related to network connectivity, and for some, privacy concerns around using breathalyzers in public, these challenges could be addressed through a combination of structural supports and tailored change planning.

Overall, couples in the *Masibambisane* study who received the mobile breathalyzer technology reported many positive sentiments and experiences including appreciation and excitement for a novel monitoring product. A major facilitator of engagement with the breathalyzers was the receipt of study support from technology navigators, power banks, cellular data vouchers, and a study phone. Other feasibility studies of breathalyzers with PLWH reported similar sentiments including that it was simple and easy to use, “cool”, “interesting”, and “fun” – despite reports of a few technical difficulties that were believed to be overcome with time and practice [23].

Although the intervention provided most participants with an engaging and enjoyable experience, there were other barriers that impacted engagement, including infrastructure, technical, and social challenges. The main challenge for completing a BAC test was persistent network and power outages. Despite problems with connectivity, over 93% of participants in the trial were satisfied with the breathalyzers and would recommend it to others [29]. However, some participants were provided with a study iPhone phone and this may have factored into reported satisfaction levels.

The BACTrack^®^ View product requires stable network connectivity to complete a BAC test. Perhaps accustomed to these realities in South Africa, people developed workaround strategies such as moving locations to an area with better coverage, using the provided power banks to make sure devices were charged before load shedding, or coming home early to do the test where cellular service may be more stable. Beyond cellular connectivity, several other challenges were noted, including difficulties with logging in (e.g., forgetting passwords), trouble connecting despite cellular connectivity, unexpected BAC readings, and concerns about privacy to complete a test in a public setting. These issues have been noted in other studies as well [23].

In many respects, couples’ descriptions of how the breathalyzer shaped their interactions around alcohol use conformed to principles of interdependence theory [25]. Specifically, interdependence theory suggests that the formation of shared goals spurs both partners’ investment in goal attainment. In the current study, this manifested as providing reminders to complete tests and carry the device, physically giving the device to a partner before leaving for drinking, warning a partner about safety precautions while carrying the phone/device, and assisting with passwords and the login process. In some instances, the inconveniences of testing led partners to leave a drinking establishment and come home (together) to test where it was safer and easier—which may have also led to reduced alcohol use.

### Clinical implications

Broadly, these findings suggest that breathalyzers may enhance dyadic coping to reduce alcohol use in couples where one or both partners engages in problematic drinking. Many couple-based treatments for substance use, including MI with couples specifically [16–18], place some emphasis on couples’ communication. It is therefore noteworthy that couples specifically indicated that use of breathalyzers facilitated communication about alcohol use outside of the intervention session. A high reading could spark a conversation within the couple to reduce drinking the next time, or a low reading could serve as positive reinforcement and help build confidence around goal achievement.

Findings from this study align with calls for the development of interventions that can address the needs of couples in which both partners use substances [37]. Almost 90% of couples in this study were dual heavy-drinking couples [29]. One important issue raised by couples was the dyadic imbalance created by providing only one partner with the breathalyzer technology, phone, data, and incentive. In future studies, the intervention might be enhanced by ensuring more equitable participation in BAC completion by both partners (where this is warranted and desired based upon clinical presentation and partner interest). The BACTrack^®^ View technology could feasibly be given to both partners. The technology is now available for Android users, which would remove bias related to the iPhone provided and minimize couple conflict.

Interestingly, some couples engaged in financial coordination around the test incentive, and others considered the economic savings that could be achieved by reducing alcohol. In resource-limited settings, the relationship between economic conditions and alcohol use is well-documented [38, 39]. Because financial concerns could be discussed during MI sessions, future iterations of the intervention might include components around alcohol and financial literacy.

### Limitations

Given the small sample of couples who received the breathalyzers, it is unclear whether other types of couples would report the same experiences. Moreover, couples who agreed to participate in couples-based MI may represent a subset with greater relationship stability, which could limit generalizability to more conflict-affected couples. Strengths include dyadic data from both partners and a combination of session and exit interview data to triangulate the themes. In sum, the breathalyzer appeared to be a multifaceted tool that, depending on individual factors and couple dynamics, could facilitate dyadic engagement around alcohol use, joint goal setting, and meaningful reductions in alcohol consumption. Innovative technical solutions (e.g., an offline feature) may be needed to maximize engagement with the breathalyzer technology in resource-poor settings with unstable power and network connectivity.

## Conclusions

Findings broadly suggest that, despite some structural and social challenges, the intervention was generally well-received. Use of the breathalyzer activated partner support and coordinated coping to reduce drinking in ways that align with interdependence theory and readily comport with the theoretical framework that undergirds MI with couples. Future studies should include a randomized controlled trial to evaluate the potential effects of the breathalyzer intervention alone and as an adjunct treatment component.

## Data Availability

Not available due to the potential to identify participants.

## Abbreviations

ART: Antiretroviral Therapy
AUDIT-C: Alcohol Use Disorders Identification Test – Consumption
BAC: Blood Alcohol Concentration
EUC: Enhanced Usual Care
HIV: Human Immunodeficiency Virus
MI: Motivational Interviewing
PLWH: People Living With HIV
SMS: Short Message Service
SSA: Sub-Saharan Africa
UCSF: University of California, San Francisco
WHO: World Health Organization
